# Affordable and effective optokinetic response methods to assess visual acuity and contrast sensitivity in larval to juvenile zebrafish

**DOI:** 10.12688/openreseurope.13923.2

**Published:** 2022-01-06

**Authors:** Alicia Gómez Sánchez, Yolanda Álvarez, Basilio Colligris, Breandán N. Kennedy

**Affiliations:** 1 Ocupharm Diagnostic Group Research, Faculty of Optic and Optometry, Universidad Complutense de Madrid, Madrid, Spain; 2 UCD Conway Institute of Biomolecular and Biomedical Research, University College Dublin, Belfield, Dublin 4, Ireland; 3 UCD School of Biomolecular and Biomedical Science, University College Dublin, Belfield, Dublin, D04 V1W8, Ireland

**Keywords:** Optokinetic response, visual acuity, spatial frequency, contrast sensitivity, visual function, zebrafish

## Abstract

The optokinetic response (OKR) is an effective behavioural assay to investigate functional vision in zebrafish. The rapid and widespread use of gene editing, drug screening and environmental modulation technologies has resulted in a broader need for visual neuroscience researchers to access affordable and more sensitive OKR, contrast sensitivity (CS) and visual acuity (VA) assays. Here, we demonstrate how 2D- and 3D-printed, striped patterns or drums coupled with a motorised base and microscope provide a simple, cost-effective but efficient means to assay OKR, CS and VA in larval-juvenile zebrafish.

In wild-type, five days post-fertilisation (dpf) zebrafish, the 2D or 3D set-ups of 0.02 cycles per degree (cpd) (standard OKR stimulus) and 100% black-white contrast evoked equivalent responses of 24.2±3.9 or 21.8±3.9 saccades per minute, respectively. Furthermore, although the OKR number was significantly reduced compared to the 0.02 cpd drum (p<0.0001), 0.06 and 0.2 cpd drums elicited equivalent responses with both set-ups. Notably, standard OKRs varied with time of day; peak responses of 29.8±7 saccades per minute occurred in the early afternoon with significantly reduced responses occurring in the early morning or late afternoon (18.5±3 and 18.4±4.5 saccades per minute, respectively). A customised series of 2D printed drums enabled analysis of VA and CS in 5-21 dpf zebrafish. The saccadic frequency in VA assays was inversely proportional to age and spatial frequency and in CS assays was inversely proportional to age and directly proportional to contrast of the stimulus.

OKR, VA and CS of zebrafish larvae can be efficiently measured using 2D- or 3D-printed striped drums. For data consistency the luminance of the OKR light source, the time of day when the analysis is performed, and the order of presentation of VA and CS drums must be considered. These simple methods allow effective and more sensitive analysis of functional vision in zebrafish.

## Plain language summary

The optokinetic response is a slow horizontal eye movement tracking an object followed by a fast movement in the opposite direction. This response can occur in humans, mammals and fish. Here, we developed simple methods to measure the optokinetic response in order to assess visual acuity and contrast sensitivity in zebrafish. In five- to 21-day-old zebrafish larvae, printed black-white or grey-white striped cylinders were rotated around the fish to induce the optokinetic response. Light levels and time of the day influence the optokinetic response. Visual acuity and contrast sensitivity can be measured simply and feasibly with printed striped cylinders from five- to 16-day-old zebrafish.

## Abbreviations

AREC: Animal Research Ethics Committee; Cd/m ^2^: candela per square meter; Cpd: cycles per degree; Cm: centimetres; CS: contrast sensitivity; Dpf: days post-fertilization; Hpf: hours post-fertilization; OKN: optokinetic nystagmus; OKR: optokinetic response; PLA: polylactic acid; VA: visual acuity; Wt: wild type.

## Introduction

The ability of researchers to effectively assess functional vision is critical to understanding the ontogeny of vision, the genetic and environmental mechanisms underlying impaired vison and the efficacy of therapeutic interventions ^1, 2^. The optokinetic response (OKR), or optokinetic nystagmus (OKN), is an innate behavioural response in humans ^3^, primates ^4^, mammals ^5^ and teleosts ^6^. In clinical practice, the OKN is an objective measure of visual acuity (VA), and can be evoked by presenting moving stimuli in front of patients by changing direction or size ^7, 8^. In natural environments, the OKR is essential for animals to hunt, feed and avoid predators. The OKR presents as a saccadic eye movement consisting of two phases: *i)* a slow eye movement following the stimulus, in the same direction as the stimulus; and *ii)* a rapid eye movement in the opposite direction to fixate on a subsequent stimulus. These movements help to stabilise the moving image presented to the retina ^9^.

Here, we sought to generate simple and affordable tools for OKR assays in zebrafish and validate their efficacy in quantifying VA and contrast sensitivity (CS) in larval and juvenile zebrafish. Zebrafish are widely used to investigate the biology of vision and blindness ^10^. Large clutches of embryos are readily obtained, which morphologically develop eyes within 24 hours, and by five days post-fertilisation (dpf) exhibit functional vision, including a robust OKR ^11, 12^. Commonly, OKR analyses in zebrafish only utilise one standard stimulus *i.e.* a drum of 0.02 cpd ( *e.g.* 1 cm width stripes) and 100% black and white contrast stripes ^13, 14^. This is not sufficient to detect subtle impairments in vision. One approach to assessing vision more thoroughly is to vary the optokinetic stimulation. By varying the width of the stripes, VA is measured efficiently ^15^. Altering the extent of contrast between stripes enables the measurement of CS ^16^. Such assays have previously been successfully performed in zebrafish by specialists often using automatic or semi-automated OKR stimulators and specialised software ^15– 17^. However, such bespoke equipment is often inaccessible or unaffordable to many research groups. Here, we describe two simple and affordable methods to assess VA and CS in zebrafish using either 2D and 3D printed striped patterns/drums to quantify OKR, VA and CS in larval and juvenile zebrafish.

## Methods

### Zebrafish husbandry

All experiments using animals were approved by ethical approvals granted by the University College Dublin Animal Research Ethics Committee (AREC-14-68-Kennedy and AREC-20-12-Kennedy). Adult wild-type ( *wt-Tübingen*) zebrafish were maintained in holding tanks on a 14:10 h light-dark cycle in a recirculating water system under environmental parameters averaging temperature of 28°C, conductivity of 1347 µS and pH of 7.1 ^18^. Adult *wt* zebrafish were fed shrimp and dry pellet food twice daily. After the noon feed, male and female adults were placed in breeding tanks and *wt* zebrafish embryos obtained by natural spawning, collected the next morning and raised in embryo medium (0.137 M NaCl, 5.4 mM KCl, 5.5 mM Na _2_HPO _4_, 0.44 mM KH _2_PO _4_, 1.3 mM CaCl _2_, 1.0 mM MgSO _4_ and 4.2 mM NaHCO _3_ with 1 ml methylene blue) until five days post-fertilisation (dpf). Larvae were fed: i) SDS 100 and paramecium from 5 to 10 dpf, ii) SDS 100, paramecium and shrimp from 11 to 20 dpf, and iii) SDS 200 and shrimp from 21 to 28 dpf. From a population of N=50 and N=40 larvae, n=12 larvae were randomly selected for VA and CS experiments at each timepoint for Protocol I and at 16 dpf for Protocol II, respectively. After each experiment, larvae were returned to the tank.

### OKR equipment

**Figure 1.**
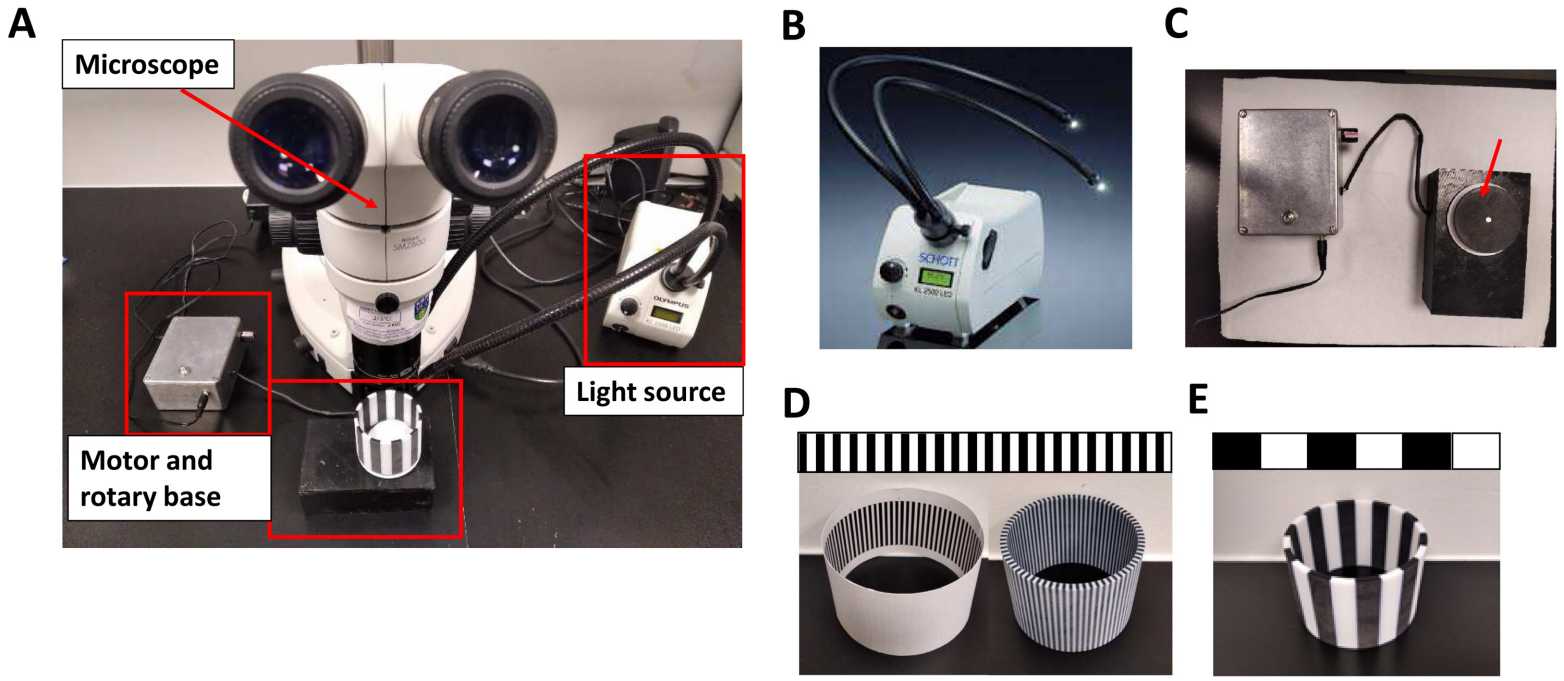
Optokinetic response equipment. **A.** Fully assembled optokinetic response apparatus. The second light guide is placed on the opposite side to the first guide to uniformly illuminate the inside of the drum. **B.** SCHOTT LED cold light source KL2500LED with fiber optic lightguide https://www.schott.com/lightingimaging/english/microscopy/index.html. Authors have obtained permission to use this image and information from SCHOTT Lighting and Imaging. **C.** Motorised rotary base to assemble the OKR drums (arrow). **D.** Optokinetic response 2D (left) and 3D (right) drums of 0.2 cycles per degree (cpd). **E.** Optokinetic response 3D drum of 0.02 (standard optokinetic response).

**Figure 2.**
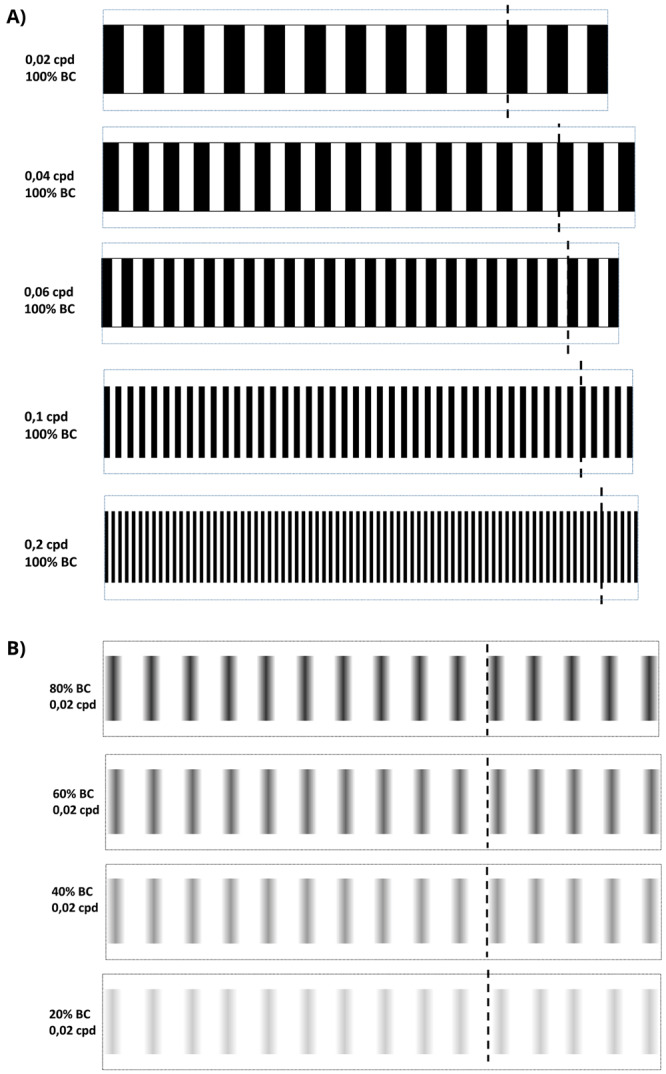
Optokinetic response 2-D visual acuity and contrast sensitivity patterns. **A**. 2D-printed visual acuity patterns. cpd = cycles per degree. **B**. 2D-printed contrast sensitivity patterns. bc = black contrast. All patterns were cut by dotted line and assembled by the discontinuous line to build the 2D-printed drums.

A simple and affordable OKR apparatus ( Figure 1) was assembled with a Nikon SMZ800 microscope (Micron Optical) to observe zebrafish eye movements; an electronic motor (RS Radionics) connected to a non-patterned 6 cm rotating circular base in which the 2D printed striped stimulus pattern was placed ( Figure 1C). A Schott KL2500 LED light source (Mason technologies) fitted with dual goose neck lightguides was positioned to illuminate inside the drum ( Figure 1B). The dimensions of the 2D-printed striped patterns, generated with MS PowerPoint® and printed on stock cardboard, were 3.4 cm high and 6 cm in diameter ( Figure 1D and Figure 2). The VA drums were 0.0277, 0.0416, 0.0638, 0.1111 and 0.2027 cycles per degree (cpd) were chosen based on a previous publication. For convenience, we will refer to these drums as 0.02, 0.04, 0.06, 0.1 and 0.2 cpd, respectively ^15^. They were designed by changing the width of the 100% black and white contrast stripes to the calculated cycles per degree (cpd = n° of cycles/360° ) when mounted on the rotating base. CS drums ranging from 100 – 20% were generated from a 0.02 cpd pattern, maintaining the spatial frequency. The black colour of the stripes was changed to include a centre to peripheral vertical gradient generated in Powerpoint™ using the “Gradient Tool, Linear Right Option” and adjusting the transparency of the Mid Gradient Stop according to the desired contrast. For example, 40% black contrast was made by changing the transparency to 60%. (C=100%-60%=40%). Additional VA drums were printed with 3D printing technology in polylactic acid (PLA), a stronger thermoplastic (Materialise UK Ltd) and placed on rotating circular base ( Figure 1E). 3D drums were designed following the same parameters as 2D-printed patterns (height= 5 cm; diameter=6 cm; cpd=0.02, 0.06 and 0.2; >99% contrast).

### Luminance measurement

An LS-100 luminance meter (Konica Minolta) measured, in candela per square meter (cd/m ^2^), the light reflected from the drum under different light intensity settings of the Schott 2500. The luminance meter was placed at 18 cm horizontally and 30 cm high from the centre of the Petri dish at 60° angle. We establish four measurements at 22.7, 12, 7 and 2%, corresponding to 3616, 1426, 769.1 and 226.7 cd/m ^2^.

### Drum velocity

The drum base was rotated with a constant angular velocity of 100 degrees per second.

### VA and CS methods (Protocols I and II)

To measure saccades/minute, a 6 cm Petri dish with 9% methylcellulose (Sigma Aldrich, UK) diluted in embryo medium was placed inside the rotating drum. From another Petri dish, larval or juvenile zebrafish in embryo medium were randomly selected and immobilised in the centre of the OKR Petri dish with 9% methylcellulose. Rotating the patterned drums 30 seconds clockwise, followed by 30 seconds counterclockwise at 100 degrees/second, evoked horizontal eye movements which were counted manually. The standard OKR utilised a drum with 0.02 cpd and 100% black-white striped contrast ( Figure 4, Figure 2A). For VA assays, the OKR was performed with 2D printed patterns of 0.02, 0.04, 0.06, 0.1 and 0.2 cpd and 100% black stripe contrast for all cpd tested ( Figure 5A, Figure 2A). In the CS assays, OKR was performed with 2D printed patterns of 100%, 80% 60%, 40% and 20% black/grey-white striped contrast, and 0.02 cpd all percentages ( Figure 5B, Figure 2B). Drums were presented following that order, from lowest to highest spatial frequency and from highest to lowest black striped contrast. Two different protocols *(I and II)* were applied. In *Protocol I*, a zebrafish larva was randomly chosen between all larvae in the dish, placed central of the 0.02 cpd drum and saccades per minute were counted. Subsequently and consecutively, the drum was replaced with one of higher spatial frequency (for VA) or lower contrast (CS) and saccades per minute counted. After completing one set of drums, another larva was randomly selected between all larvae in the dish and used to repeat the same drum sequence. In *Protocol II*, instead of presenting each drum of a series to the same larva, different specimens were used for each drum, i.e. larvae were naïve for OKR. In practice, a larva was analysed with 0.02 drum, saccades per minute were counted and then replaced by another larva which was subjected again to the same drum. This procedure was repeated for the rest of the drum patterns.

### Statistical analysis

Statistical analysis was completed using GraphPad Prism 7.00 software (GraphPad, San Diego, CA). All saccades per minute values described in this manuscript are a group average. One-way repeated measures ANOVA was employed to determine significant differences between groups followed by Bonferroni’s multiple comparisons test except for Figure 6B and Figure 7B where One-way unpaired ANOVA was used to determine significant differences between groups followed by Tukey’s multiple comparisons test. Significance levels were set at p < 0.05.

An earlier version of this article can be found on bioRxiv (doi: https://doi.org/10.1101/2021.04.26.441419).

## Results

### 2D and 3D printed VA patterns elicit equivalent OKR responses in 5 dpf zebrafish

The manual OKR equipment set-up ( Figure 1) permits simple exchange of stimulus patterns to measure VA. This apparatus was assembled using a microscope ( Figure 1A) to observe zebrafish eye movements, a light source ( Figure 1B) and an electronic motor connected to a 6 cm rotating circular base ( Figure 1C). 2D or 3D printed stimulus drums ( Figure 1D) were placed on the circular base which was rotated electronically to evoke eye movements. A standard OKR pattern of 0.02 cpd (100% contrast) ( Figure 1E) and customised 0.06 and 0.2 cpd patterned stimuli ( Figure 1D) were produced by 2D or 3D printing (see Methods and Figure 2 for full details on OKR assembly).

**Figure 3.**
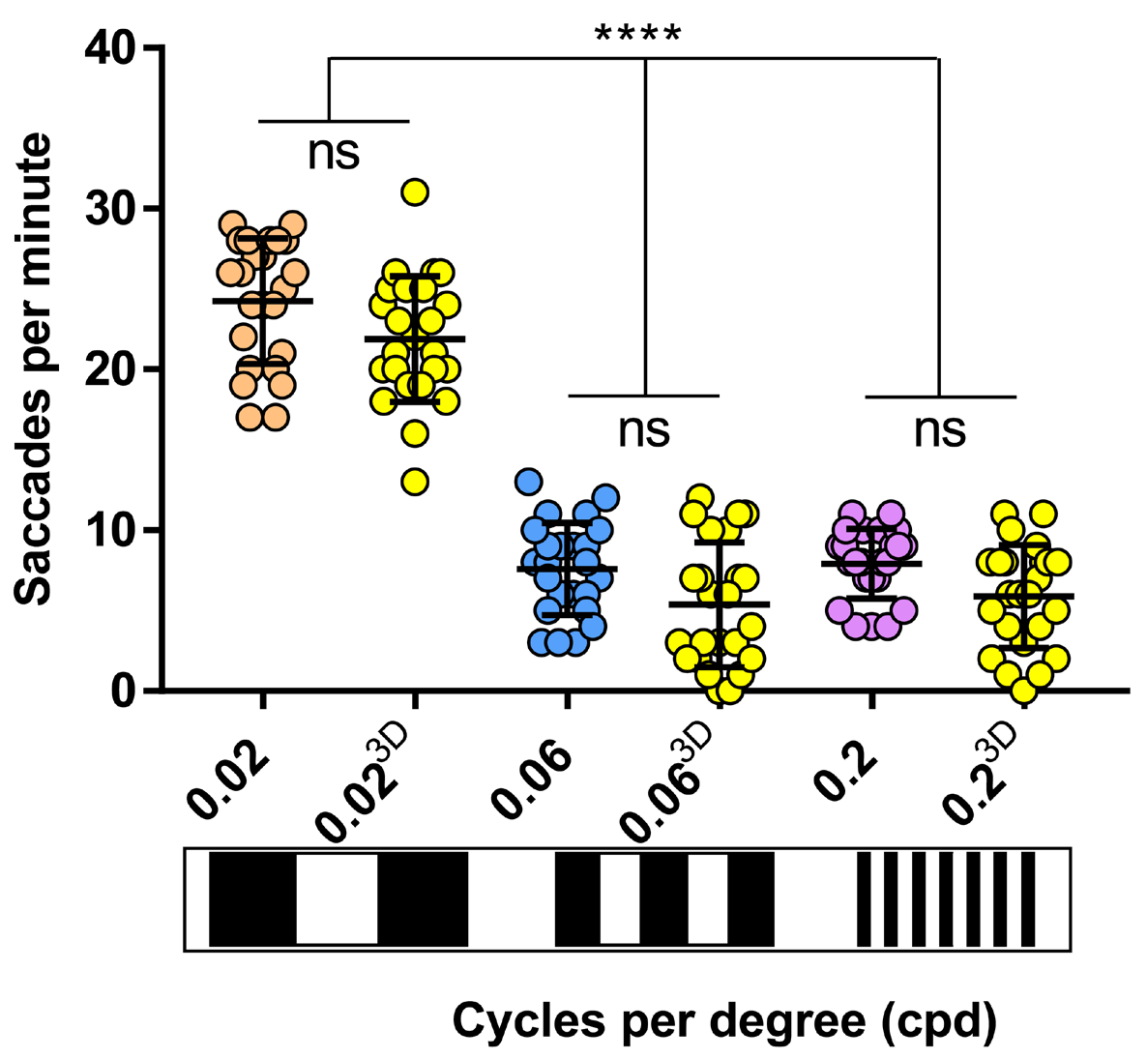
2D drums evoke same saccades frequency as 3D drums. Visual acuity responses obtained with 3D drums (yellow dots) don't vary with respect of cardboard-printed drums (0.02 cpd, orange dots; 0.06 cpd, blue dots; 0.2 cpd, purple dots). 0.06 and 0.2 cpd responses evoked with 2D and 3D-printed drums were significant lower than standard optokinetic response activity (0.02 cpd) with 2D and 3D-printed drums. Data were analyzed by one way ANOVA and Bonferroni’s multiple comparison test, where ns is no significant difference (p>0.05) and ****=p<0.0001. Error bars indicate standard deviation. Midline of error bars represents the group average. Three replicates of eight larvae per drum, n=24.

VA analysis with 2D and 3D printed drums was performed on 5 dpf zebrafish larvae (~123.5 hours post-fertilisation - hpf) using *Protocol I* (see Methods) ( Figure 3). The OKRs evoked by the 3D and 2D-printed drums were equivalent. More specifically, the OKR activity with the 0.02 cpd 2D-printed pattern (24.2±3.9 saccades per minute) was equivalent to the 21.8±3.9 saccades per minute evoked with the 0.02 cpd 3D-printed drum ( Figure 3). Similarly, there was no significant difference between the 7.5±2.9 and 5.3±3.9 saccades per minute produced by the 0.06 cpd 2D and 3D-printed drum, respectively ( Figure 3). At the highest spatial frequency tested, 0.2 cpd, the number of saccades evoked by the 2D (7.9±2.2 saccades per minute) and 3D (5.8±3.2 saccades per minute) drums also showed no significant difference. Therefore, both 2D and 3D printed drums can be used to measure the VA of 5 dpf zebrafish larvae, the 3D-printed drums offering a more durable, but more costly option.

### The zebrafish larval OKR response is modulated by time of day and luminance levels

**Figure 4.**
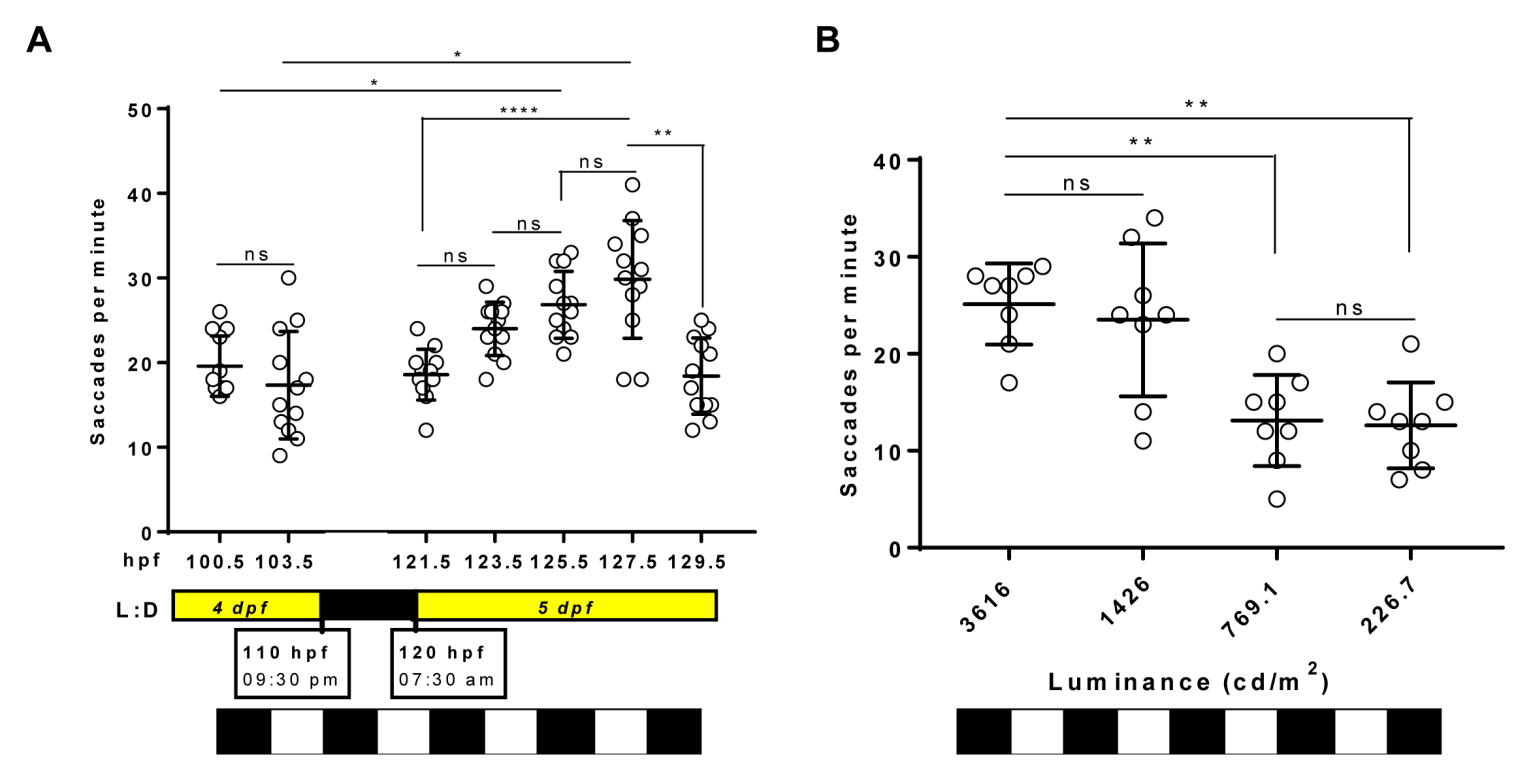
Optokinetic response (OKR) is modulated by time of day and luminance levels. **A.** Standard OKR (0.02 cycles per degree [cpd], 100% black contrast, 3616 candelas per square meter [cd/m ^2^]) at different timepoints along 4 and 5 days post fertilisation (dpf). Equivalent times (100.5 vs. 125.5 hpf; 103 vs. 127.5 hpf) show an increase in OKR between 4 and 5 dpf. Highest OKR yields at 127.5 hpf. **B.** Standard OKR (0.02 cpd, 100% black contrast) at different levels of luminance at 125 hpf. Higher levels of luminance evoked a better response on zebrafish but at 1426 cd/m2, OKR response is more variable (SD=7.8 saccades per minute) than 3616 cd/m2 (SD=4.1 saccades per minute). Data were analyzed by one-way ANOVA and Bonferroni’s multiple comparison tests, where ns is no significant difference, (p>0.05), **=p<0.01 and ****=p<0.0001. Error bars indicate standard deviation (SD). Midline of error bars represents the group average. One replicate of 12 larvae per timepoint, n=12.

To determine if the zebrafish larval OKR has diurnal variations, the number of saccades generated with the standard 3D-printed OKR drum (0.02 cpd) was determined at seven timepoints distributed throughout the light phases of the standard 14-hour light: 10-hour dark cycle ( Figure 4A). At 5 dpf, the trend observed was an increasing number of saccades until the afternoon with a subsequent drop in response ( Figure 4A). The highest OKR (29.8±7 saccades per minute) was observed at early afternoon/127.5 hpf, which was significantly higher (p=0.0001) than the OKRs observed at early morning/121.5 hpf (18.5±3 saccades per minute) or at late afternoon/129.5 hpf (18.4±4.5 saccades per minute). The midday and early afternoon responses on 5 dpf (125.5 and 127.5 hpf, respectively) were significantly greater than the corresponding time of day responses at 4 dpf (100.5 and 103.5 hpf, respectively).

To evaluate if the 5 dpf OKR behaviour varied with brightness intensities, the standard OKR was assessed under luminance ranging from 226.7–3616 cd/m ^2 ^( Figure 4B) at 125 hpf. The largest OKR activity occurred at 3616 and 1426 cd/m ^2^ (25.1±4.2 and 23.5±7.9 saccades per minute, respectively). The responses at 769.1 and 226.7 cd/m ^2^ (13.1±4.7 and 12.6±4.4 saccades per minute, respectively) were significantly lower (p=0.0081 and p=0.0035, respectively) than at 3616 cd/m ^2^. In summary, the larval OKR shows response variations based on time of day recorded and light intensity used.

### The 2D/3D-printed striped patterns enable discrimination of VA and CS in larval zebrafish

**Figure 5.**
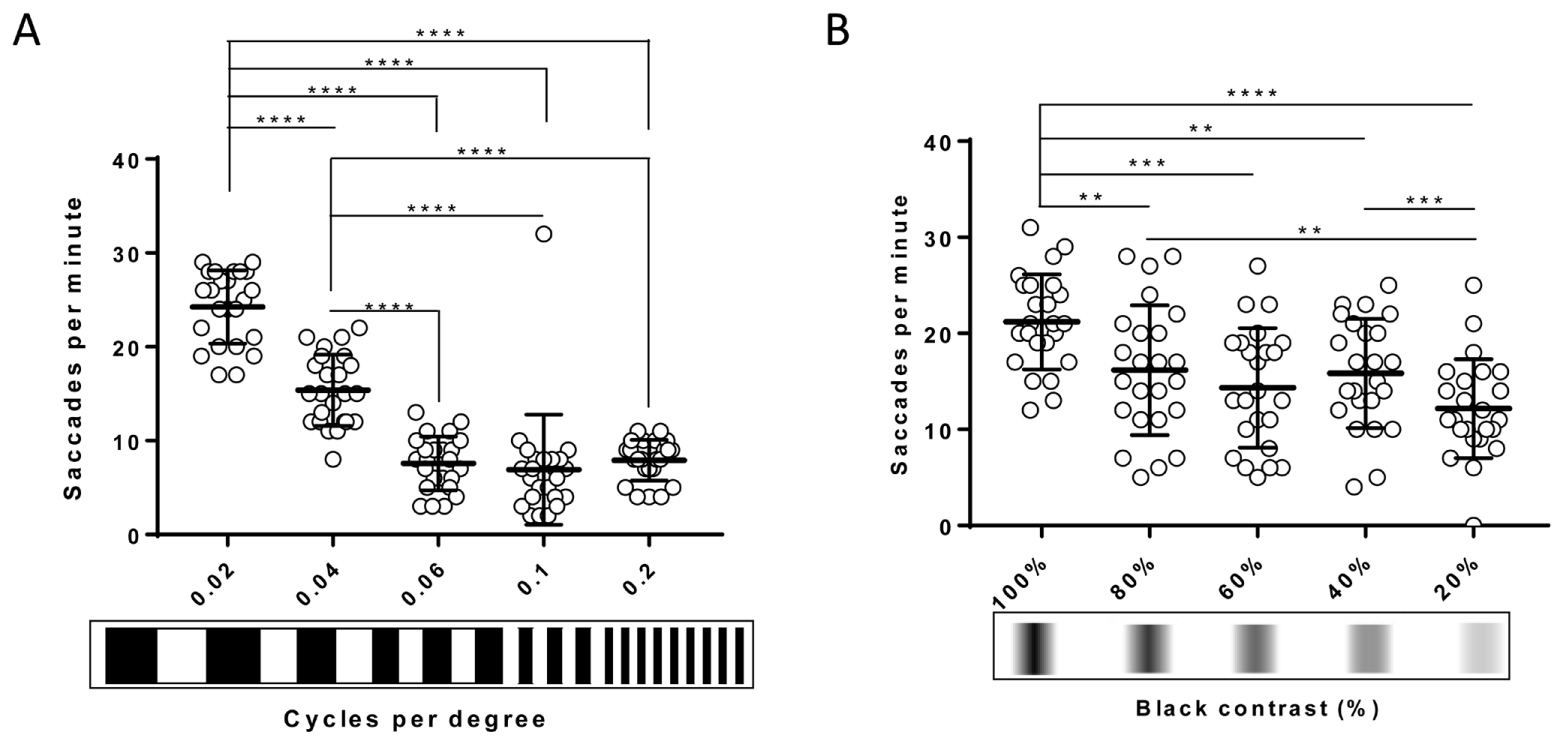
The 2D-printed drums enable discrimination of visual acuity and contrast sensitivity in larval zebrafish. **A.** Visual acuity of 5 dpf wild-type zebrafish larvae decreases progressively when width of the stripes is reduced compared to standard optokinetic response (OKR) and 0.04 cpd. From 0.06 cpd, response is constant. **B.** Contrast sensitivity of 5 dpf wild-type zebrafish larvae decreases slowly when contrast between black-white stripes is lowered compared to standard OKR. 20% black contrast evokes the lowest response. Data were analyzed by repeated measures one-way ANOVA and Bonferroni’s multiple comparison test, where ns is no significant difference, (p>0.05), **=p<0.01, ***=p<0.001 and ****=p<0.0001. Error bars indicate standard deviation. Midline of error bars represents the group average. Three replicates of eight larvae, n=24 measurements per pattern.

Establishment of affordable VA and CS assays offers researchers the potential to identify more subtle defects in zebrafish vision than using standard OKR drums. Thus, bespoke 2D-printed striped patterns of 0.04 and 0.1 cpd for VA were generated (see Methods for details) and tested ( Figure 5). At 123 hpf, using *Protocol I* (see Methods), an increased number of stripes reduced the number of saccades per minute, but robust and reproducible responses were observed at each cpd tested ( Figure 5A). At 0.04 cpd, the OKR activity (15.3±3.8 saccades/minute) was significantly (p<0.0001) lower compared to the standard OKR pattern of 0.02 cpd (24.2±3.9 saccades per minute), but significantly higher than the response with the 0.06 cpd pattern (7.6±2.9 saccades per minute, p<0.0001). The average saccades per minute with the 0.06 cpd pattern (7.6±2.9 saccades per minute) is similar to the 0.1 and 0.2 cpd pattern (6.9±5.9 and 7.9±2.2 saccades per minute, respectively).

CS assays were also performed using 2D printed drums and *Protocol I* at 125 hpf ( Figure 5B). The OKR activity evoked by the 0.02 cpd patterns with decreasing contrast was significantly reduced (80%, p=0.0022; 60%, p=0.0001; 40%, p=0.004, and 20%, p<0.0001) compared to the standard OKR drum of 0.02 cpd and 100% contrast. For example, at 80% black-white contrast, the 16.1±6.7 saccades per minute were significantly lower (p=0.0022) than the 21.2±4.9 saccades per minute evoked with the standard OKR drum pattern (0.02 cpd). There were no significant differences in response between the 80% contrast pattern and the 60% or 40% contrast pattern. The response from the 20% contrast pattern (12.2±5.1 saccades per minute) was significantly lower than with the 80% and 40% contrast pattern (p=0.0091 and p=0.0003, respectively. In summary, the 2D-printed patterns provide a simple and affordable method to assess CS and VA assays in zebrafish larvae.

### The zebrafish VA response shows age-dependent variations

**Figure 6.**
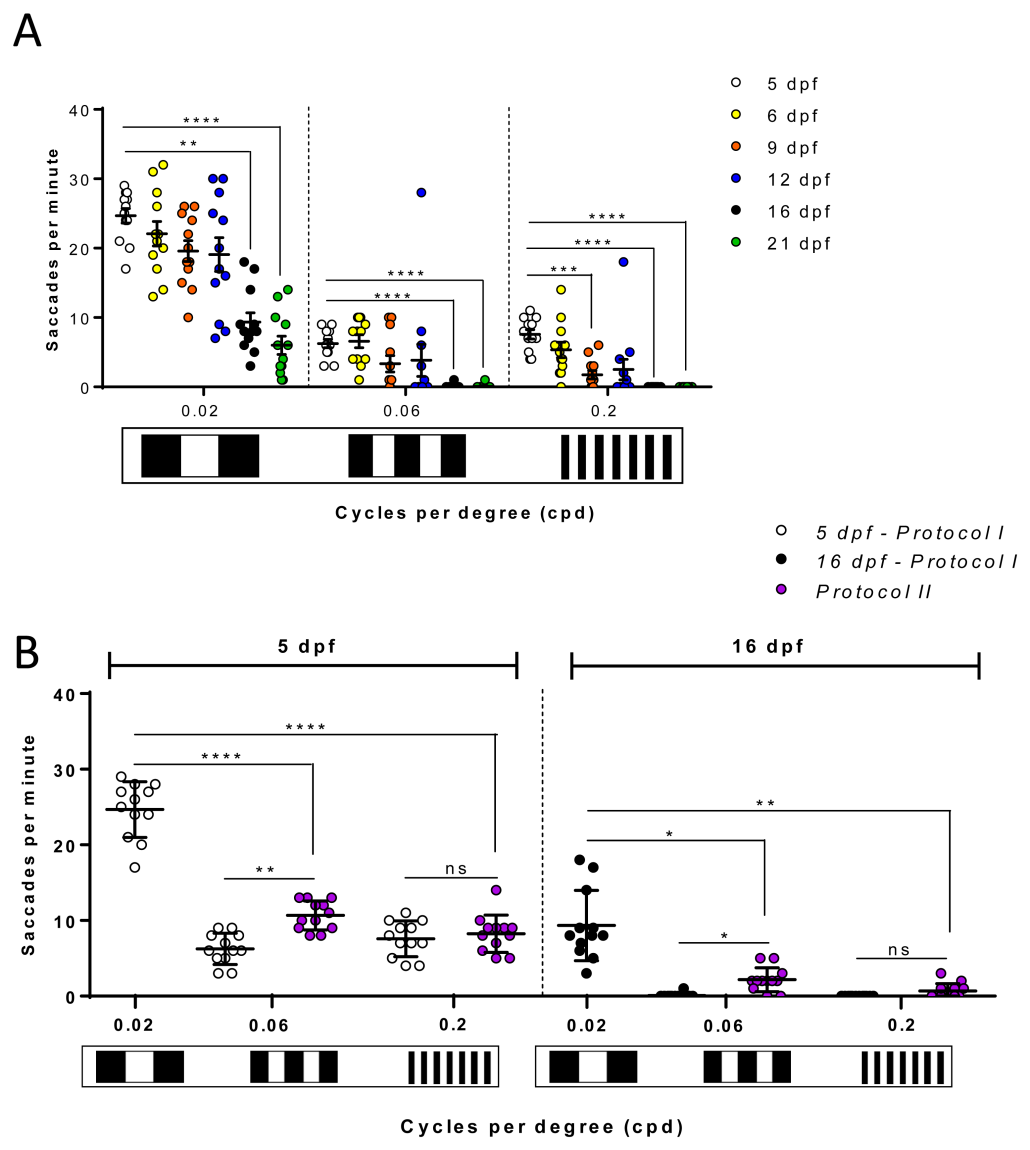
Zebrafish visual acuity responses show age-dependent variations. **A.** Visual acuity of zebrafish from 5 to 21 days post fertlisation (dpf) drops significantly from 16 dpf following *Protocol I* at 0.02, 0.06 and 0.2 cpd. At 0.2 cpd, this decreased response is also remarkable on 9 dpf. One replicate of 12 larvae per each set of patterns. **B.** Comparison of visual acuities measured with *Protocol I* (black dots) and *Protocol II* (red dots) on 5 dpf and 16 dpf. 0.02 cpd responses at 5 dpf (purple dots) and 16 dpf (white dots) belong to *Protocol I* and *Protocol II* as it is the first pattern tested. There is no difference between both protocols except at 0.06 cpd where 5 dpf naïve larvae showed a higher number of saccades. Data were analyzed by repeated measures one-way ANOVA and Bonferroni’s multiple comparison test in **A** and unpaired measures one-way ANOVA and Tukey’s multiple comparison test in **B**. In both statistical tests, ns is no significant difference, (p>0.05), **=p<0.01, ***=p<0.001 and ****=p<0.0001. Error bars indicate standard error of mean in **A** and standard deviation in **B**. Midline of error bars represents the group average. One replicate of 12 independent larvae for each pattern, n=12.

Using the 2D-printed patterns, we determined if the OKR-based VA response varies with age in larval to juvenile zebrafish aged 6, 9, 12, 16 or 21 dpf at 123 hpf. Interestingly, with *Protocol I* the measured VA responses decreased with age ( Figure 6A) for all tested patterns. The largest OKR of 24.6±3.7 saccades per minute was achieved at 5 dpf with a pattern of 0.02 cpd frequency ( Figure 6A). The lowest OKR, with absence of any saccadic eye movements (0±0 saccades per minute), was obtained with 16 and 21 dpf zebrafish using patterns of 0.2 cpd ( Figure 6A). With patterns of 0.02 cpd, the OKR was significantly reduced at 16 dpf (p=0.0016) and 21 dpf (p<0.0001) compared to 5 dpf larvae, with 62% and 76% reductions in eye saccades, respectively. With patterns of 0.06 cpd, the highest responses were observed at 5 and 6 dpf (6.2±2.1 and 6.5±3.2 saccades per minute, respectively), which significantly declined at 16 dpf (0.1±0.3 saccades per minute, p<0.0001) and 21 dpf (0.1±0.3 saccades per minute, p<0.0001) compared to 5 dpf. For the highest VA patterns tested (0.2 cpd, with highest number of stripes), the largest OKR was observed in 5 dpf larvae (7.5±2.4 saccades per minute) and significantly reduced responses were observed in 9 (1.7±2.1 saccades per minute, p=0.0004), 16 (0±0 saccades per minute, p<0.0001) and 21 (0±0 saccades per minute, p<0.0001) dpf zebrafish. Note that at 16 dpf, when responses to VA and CS drums dropped, fish immobilisation in methylcellulose during drum stimulation was more difficult compared to earlier stages. In addition to observing an age-dependent reduction in OKR at each cpd frequency, we also observed that the level of response with the 0.06 and 0.2 cpd patterns were much lower than with the 0.02 cpd standard drum ( Figure 6A). In *Protocol I,* the data is generated based on first testing larvae at the lowest spatial frequency, and subsequent testing in the next higher spatial frequency drum. Therefore, to assess whether the reduction in OKR with drums of higher spatial frequency was due to adaptation to previous OKR stimuli, we repeated the assays on 5 and 16 dpf at 123 hpf, using *Protocol II* (see Methods for details) where each fish was tested with only one drum pattern ( Figure 6B). In Figure 6b, datasets from 5 and 16 dpf groups for Protocol I are from the same groups in Figure 6a. In 5 dpf zebrafish, there was no significant difference in OKR using *Protocol I* or *II* for 0.2 cpd pattern ( Figure 6B). There was a significant increase (p=0.0017) in OKR of 5 dpf larvae with *Protocol II* compared to *Protocol I* with the 0.06 cpd pattern ( Figure 6B). However, the *Protocol II* response of 10.6±1.9 saccades per minute with the 0.06 cpd pattern was still significantly lower (p<0.0001) than the 24.6±3.7 saccades per minute observed under *Protocol I* with the 0.02 cpd standard drum ( Figure 6B). In 16 dpf zebrafish, a slight but significant increase (p=0.044) in OKR was noticed when *Protocol II* is compared to *Protocol I* with the 0.06 cpd pattern. With the 0.2 cpd pattern and 16 dpf zebrafish there was no significant difference using *Protocol I* or *Protocol II*. At 16 dpf, the 0.06 cpd response obtained with *Protocol II* (2.1±1.6 saccades per minute) is significantly lower (p=0.0204) than the 0.02 cpd response (9.3±4.6 saccades per minute). In summary, all the above suggests that VA measurements drop after 12 dpf. Additionally, care needs to be taken regarding a consistent order of testing the VA drums to avoid experimental artifacts.

### The zebrafish CS responses show age-dependent variations

**Figure 7.**
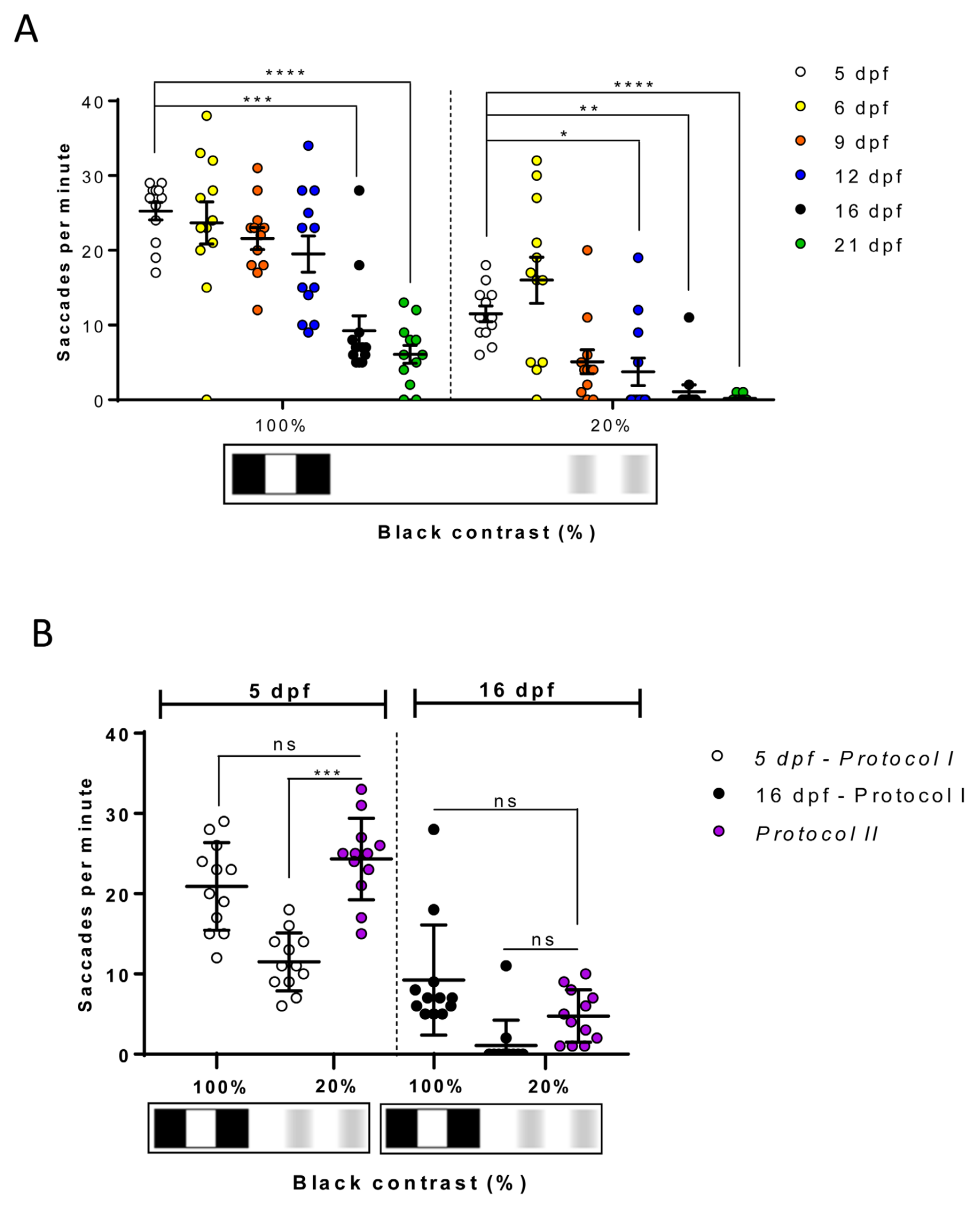
The contrast sensitivity response of juvenile zebrafish diminishes with age. **A.** Optokinetic response to 20% is significant at 12, 16 and 21 days post fertilisation (dpf). **B.** Comparison of contrast sensitivity responses measured with *Protocol I* (black dots) and *Protocol II* (red dots). 100% black contrast at 5 dpf (purple dots) and 16 dpf (white dots) belong to *Protocol I* and *Protocol II* as it is the first pattern tested. Responses of 20% black contrast with *Protocol II* are higher than when evoked with *Protocol I*. However, there are no differences between both protocols at 16 dpf. Data were analyzed by repeated measures one-way ANOVA and Bonferroni’s multiple comparison test in **A** and unpaired measures one-way ANOVA and Tukey’s multiple comparison test in **B**. In both statistical tests, ns is no significant difference, (p>0.05), **=p<0.01, ***=p<0.001 and ****=p<0.0001. Error bars indicate standard error of mean in **A** and standard deviation in **B**. Midline of error bars represents the group average. One replicate of 12 independent larvae for each pattern, n=12.

Subsequently, we determined if the CS responses obtained using the 2D printed patterns displayed age-dependent variations. At 125 hpf, using *Protocol I* and 0.02 cpd drums with 100% black-white contrast, the largest response of 25.2±4.1 saccades per minute was observed with 5 dpf larval zebrafish ( Figure 7A). Responses to these drums showed significant reduction with age, but reproducible visual behaviour responses were still observed with 16 and 21 dpf juvenile zebrafish (9.2±6.9 saccades per minute, p=0.0007; and 6±4.2 saccades per minute, p<0.0001, respectively). Similarly, with the 20% contrast drums, the largest responses were observed with 5 and 6 dpf (11.5±3.6 and 16±10.7 saccades per minute, respectively) larvae. Numbers declined with age and significant reductions were observed in 12, 16 and 21 dpf juveniles (3.7±6.3 saccades per minute, p=0.04; 1.1±3.2 saccadic per minute, p=0.0019; 0.1±0.4 saccades per minute, p<0.0001, respectively). As mentioned earlier, fish immobilisation and saccade counting in older fish is less consistent. Again, at 125 hpf, we utilised *Protocol II* to determine if reduced responses were due to desensitisation to consecutive stimuli. In Figure 7B, datasets from 5 and 16 dpf groups for *Protocol I* are from the same groups in Figure 7A. In 16 dpf zebrafish, there was no significant difference in OKR using *Protocol I or II* when testing 20% contrast drums ( Figure 7B). In 5 dpf zebrafish, there was a significant increase (p=0.0002) in OKR at 20% contrast when *Protocol II* is compared to *Protocol I* ( Figure 7B). Indeed, the *Protocol II* response of 24.3±5.1 saccades per minute with the 20% contrast drum is equivalent to the response observed under *Protocol I* with 100% contrast ( Figure 7B), suggesting the diminished CS response is due to desensitisation. In summary, our data suggests that CS responses decrease significantly after 9 dpf, and highlight the importance of strict consistency to be taken while testing different CS patterns on the fish to avoid confounding.

## Discussion

### Affordability and accessibility

The OKR is a strong, innate visual behaviour that is very useful to characterise functional vision in zebrafish ^12^. We employed 2D and 3D printed patterns, of different stripe width or different black-white contrast, to effectively and affordably assay OKR, VA and CS in 5 to 21 dpf zebrafish. The remaining equipment required is accessible and affordable as suitable microscopes are commonly available in laboratories and the other components *e.g.* motor, light source and 2D/3D-printed patterns can be acquired easily and cost-effectively. While 2D-printed can be easily printed on cardboard, they are less durable so can be used for pilot experiments. However, if researchers prefer a more durable option, 3D-printed drums are still an affordable option (less than 100€ per drum) yielding equivalent results as 2D-printed drums. Whilst automated or computerised devices were previously used to report OKRs, those systems have high costs (up to €30,000), prohibitive to many research groups. Furthermore, computerised measurements of OKR, VA and CS, apply software to disaggregate the collective saccadic eye movement into eye velocity, gain or amplitude parameters ^6, 15, 16^. This requires establishment of thresholds based on algorithms and formulas using specialist programmes ^6, 15^. In summary, the manual OKR set-up described here enables refined and accurate evaluation of OKR, VA and CS in zebrafish larvae, it is easy to use, does not require specialist software and is up to 10 times more affordable.

### Effectiveness and sensitivity

With the 2D and 3D printed patterns, the magnitude of the 5 dpf VA response progressively decreased from the 0.02 cpd (standard OKR) to the 0.2 cpd (finest stripe width tested) pattern. This inverse relationship between saccadic response and stripe width agrees with previous studies using digitalised OKR set-ups and a 0.02 – 0.2 cpd range of VA patterns ^15, 16^. Notably, those studies, which pre-stimulated the larvae with a 0.06 cpd pattern before testing, reported 0.16 cpd as the highest VA pattern to evoke an OKR in 5 dpf larvae ^15, 16^. However, with our 2D and 3D printed drums, an even finer VA stimulus of 0.2 cpd elicits reproducible OKRs of 5.8±3.2 – 7.9±2.2 saccades per minute, providing enhanced ability to identify more subtle visual impairment phenotypes.

### Diurnal variability

There is clear evidence of dynamic anatomical and behavioural development of zebrafish vision up to 5 dpf ^9, 19– 21^. A previous analysis of diurnal variations in OKR at 5 dpf ^22, 23^ showed no difference in the number of saccades evoked during the day at 122 hpf (early morning; 27 saccades per minute) and 134 hpf (early evening; 25 saccades per minute), but dropping to 0 saccades per minute at 137 hpf (night) ^22^. Another study performed diurnal OKR analysis at different timepoints ^23^, wherein at 125 hpf the OKR gain peaked and then decreased progressively at 129 and 133 hpf. Here, we investigate the OKR between 4 and 5 dpf using even shorter time intervals. We found a cyclic modulation of OKR activity from a base of 19.5±3.6 saccades per minute at 100.5 hpf (midday ) at 4 dpf, reaching a peak of 29.8±7 saccades per minute at 127.5 hpf (early afternoon) on 5 dpf and then troughing at 18.4±4.5 saccades per minute at 129.5 hpf (late afternoon) on 5 dpf. The peak responses at 125.5 and 127.5 hpf (26.8±4 and 29.5±7 saccades per minute) and diminished response at 129.5 hpf (18.4±4.5 saccades per minute) are consistent with Huang *et al.* ^23^. This diurnal variation may be attributed to circadian rhythms that drive diurnal and nocturnal behaviours in zebrafish ^23, 24^. In summary, a more extensive characterization at shorter times post-fertilization demonstrates significant diurnal variations in the OKR and highlights the importance of carefully controlling the time of day when OKR analysis is performed.

### Light variability

OKR gain, the ratio between eye velocity and stimulus velocity during the slow saccadic phase, was previously reported to increase with luminance from 0.38 cd/m ^2^ up to 388 cd/m ^2^ levels ^16^. Here, we demonstrate that higher luminance levels of 769.1 to 3616 cd/m ^2^ increase the saccadic frequency from 13.1±4.7 to 25.1±4.2 saccades per minute. Notably, we did not, as in previous studies, measure the luminance from where the stimulus was projected ^16^. Instead our luminance was measured at the position of the fish in the methylcellulose to measure the ambient illumination surrounding the fish more accurately. In summary, the luminance of the light source must be measured and controlled during all analyses to avoid this confounding variable which affects saccadic frequency.

### Contrast sensitivity and visual acuity detection

Previous VA studies on 5 dpf larvae report that the magnitude of the OKR gain or eye velocity between 0.02 and 0.2 cpd was indirectly proportional to spatial frequency ^15, 16^. More specifically, an eye velocity of 4 degrees/second at 0.05 cpd reduced to 0 degrees/second (no eye movements) at 0.2 cpd ^12^. At the highest drum velocity tested (22.5 degrees per second) a gain response of 0.2 (max. gain=1) at 0.02 cpd decreased to 0.025 at 0.16 cpd; however, the gain peak of 0.3 was reported at a mid-frequency of 0.06 cpd ^16^. The VA responses with the 2D printed patterns concur with this spatial frequency-dependency as evidenced by 24.2±3.9 saccades per minute at 0.02 cpd reducing to 7.9±2.2 saccades per minute at 0.2 cpd, the latter response contrasting with no eye movements using the computerised OKR hardware. Thus, the OKR set-up described here emulates VA responses of automatic devices ^15, 16^ and furthermore, it detects quantifiable responses at higher spatial frequencies ^12, 13^.

CS analysis using the 2D-printed drums (20-100%) at 5 dpf show a similar trend, as previously reported with computerised set-ups (0.7 to 100% contrast). A higher number of OKR saccades or greater OKR gain is observed as the black-white contrast increases ^16, 25^. Notably, these computerised devices reported a low gain ^16^ and no eye movements ^25^ at 20% black-white contrast. However, our manual OKR set-up evokes reproducible OKR saccades of 12.1±5.1 per minute at the 20% black-white contrast. Hence, our affordable 2D-printed drums can elicit OKR that discriminate higher VA frequencies and lower black-white contrast, enabling more sensitive detection of VA and CS in 5 dpf zebrafish.

### Age variability

OKR analysis in juvenile zebrafish older than 5 dpf was previously reported using computerised OKR set-ups ^6, 12, 16^. Orger *et al.* ^12^ used a lower drum velocity (10 degrees per second) and described the standard OKR activity at 7 dpf, showing robust eye saccades through a motion detection OKR. Beck *et al.* ^6^ investigating the OKR phases from 5 to 35 dpf, found that at 50 degrees per second drum velocity, gain decreased in all tested ages (5 to 35 dpf). Here, we use the 2D-printed drums to describe the saccadic frequency of 5 to 21 dpf zebrafish based on spatial frequency ( Figure 6A). For all ages, saccadic frequency decreased as spatial frequency increased. We obtained quantifiable responses at all tested ages except at 16 and 21 dpf using our 0.06 and 0.2 cpd patterns. This reduction when zebrafish were older, was also observed using 100% and 20% black-white contrast 2D-printed patterns in 5 to 21 dpf zebrafish ( Figure 7A). At 6 dpf, Rinner *et al.* ^16^ previously reported that OKR gain with 100% black-white contrast was approximately 0.7, decreasing to 0.3 with 20% black-white contrast. This response is similar to what we obtained at 6 dpf using the 2D-printed patterns, where 23.6±9.8 saccades per minute were obtained at 100% black-white contrast, decreasing to 16±10.7 saccades per minute at 20% black-white contrast. In summary, our data suggests that manual VA/CS analysis, using 2D-printed patterns, can be used to detect spatial frequency and contrast discrimination by zebrafish larvae at 6, 9, 12, 16 and 21 dpf. 

### Protocol variability/desensitisation

The significant drop of VA and CS response observed after 16 dpf may be explained by the use of methylcellulose to immobilise the larvae, but which is also reported to hamper oxygen exchange in zebrafish older than 7 dpf and to decrease the OKR gain ^9^.

Studies on adult zebrafish, aged between 4–16 ^26^ and 12–24 ^17^ months, placed the fish further from the stimulus, *i.e.* 7.3 cm ^26^ and 19.5 ^17^ cm *versus* the 3 cm used here. According to the VA concept and VA examinations in children ^27^, the eye to stimulus distance should be increased with age, which suggests that 16 dpf could be a *“key”* time-point to increase the distance stimulus-eye in zebrafish.

We considered that habituation could also account for reduced VA/CS at older stages. However, using 16 dpf naïve larvae ( *Protocol II*), responses at highest spatial frequencies (0.2 cpd) and lowest contrast (20% black-white contrast) were similar as those tested in 16 dpf in *Protocol I*. Our overall interpretation is that, in general, *Protocol I* is more suitable to conduct VA/CS studies in zebrafish larvae due to ethical considerations to reduce the number of individuals used, while enabling follow-up of VA or CS multiple data obtained from a single specimen. *Protocol II* could be more suitable, however, if obtaining maximum responses at 5 dpf is relevant for the study.

## Conclusions

The OKR set-up described here can be easily and cost-effectively acquired to measure OKR. Our 2D-printed patterns can reliably and feasibly quantify VA and CS response in zebrafish larvae from 5 to 16 dpf. The age of the fish used, the time of the day the assay performed, the light levels within the fish position and pre-stimulation can vary the OKR and must be accurately determined for a consistent OKR, VA and CS analysis. The 2D/3D drums and methods described here can be utilised to identify and characterise more effectively zebrafish models with visual deficits.

## Data availability

### Underlying data

Zenodo: Affordable and Effective Optokinetic Response Methods to Assess Visual Acuity and Contrast Sensitivity in Larval to Juvenile Zebrafish. https://doi.org/10.5281/zenodo.5146212 ^28^.

This project contains the following underlying data:

- Fig. 3 - 2D drums evoke same saccades frequency as 3D drums.csv
- Fig. 4A - OKR Response is Modulated by Time of Day.csv
- Fig. 4B - OKR Response is Modulated by Luminance Levels.csv
- Fig. 5A - The 2D-printed Drums Enable Discrimination of Visual Acuity in Larval Zebrafish.csv
- Fig. 5B - The 2D-printed Drums Enable Discrimination of Contrast Sensitivity in Larval Zebrafish.csv
- Fig. 6A - The Zebrafish Visual Acuity Responses Shows Age-Dependent Variations.csv
- Fig. 6B- The Zebrafish Visual Acuity Responses Shows Age-Dependent Variations.csv
- Fig. 7A - The Contrast Sensitivity Response of Juvenile Zebrafish Diminish with Age.csv
- Fig. 7B - The Contrast Sensitivity Response of Juvenile Zebrafish Diminish with Age.csv

### Reporting guidelines

Zenodo: ARRIVE checklist for “Affordable and effective optokinetic response methods to assess visual acuity and contrast sensitivity in larval to juvenile zebrafish”. https://doi.org/10.5281/zenodo.5118706 ^29^.

Data are available under the terms of the Creative Commons Attribution 4.0 International license (CC-BY 4.0).
