# Supplementary material for: Affordable and effective optokinetic response methods to assess visual acuity and contrast sensitivity in larval to juvenile zebrafish
Source: Open Res Eur. 2022 Jan 6;1:92. Originally published 2021 Aug 12. [Version 2] doi: 10.12688/openreseurope.13923.2 (PMC10446059; doi:10.12688/openreseurope.13923.2)
Supplement: Peer review history

# Peer review: Reviewer response for version 2

**DOI:** 10.21956/openreseurope.15543.r28281

**Published:** 2022-01-11

**Authors:** Logan Ganzen

**Affiliations:** 1 UC Berkeley, Berkeley, CA, USA

The authors have sufficiently addressed my proposed concerns in this revision.

Is the rationale for developing the new method (or application) clearly explained?

Yes

Is the description of the method technically sound?

Yes

Are the conclusions about the method and its performance adequately supported by the findings presented in the article?

Partly

If any results are presented, are all the source data underlying the results available to ensure full reproducibility?

Yes

Are sufficient details provided to allow replication of the method development and its use by others?

Partly

Reviewer Expertise:

My area of research is generally in visual neuroscience. My doctoral research involved the development of zebrafish behavioral assays to perform drug screening on zebrafish models of disease.

I confirm that I have read this submission and believe that I have an appropriate level of expertise to confirm that it is of an acceptable scientific standard.

# Peer review: Reviewer response for version 1

**DOI:** 10.21956/openreseurope.15007.r28060

**Published:** 2021-11-22

**Authors:** Ralph Nelson

**Affiliations:** 1 National Taiwan University, Bethesda, MD, USA

Rotating striped drums are not a new technique to evoke an optokinetic response (OKR) from zebrafish eyes. The reviewer’s first encounter with this technique was Brokerhoff *et al. *(1995) ‘ *A behavioral screen for isolating zebrafish mutants with visual system defects*’. This design remains quite viable. This article provides a lot of details enabling labs to set up such a system, inexpensively as the authors emphasize. The article also gives a broad range of constraints to consider in experimental design. The reviewer was particularly enlightened by the studies of circadian rhythm. The larvae are most responsive at midday or early afternoon. Older larvae don’t perform well in these protocols. The authors are happy to point out that the inexpensive rotating-drum stimulus was significantly more effective than expensive computer-driven displays at evoking OKR responses and extended the range of contrasts and spatial frequencies that could be examined. The reviewer wants to add that in his lab one of his students had little trouble making her own computer driven OKR stimulus for zebrafish larvae. This was a laptop, one that folds out flat, with rotating pinwheels generated by PsychoPy toolbox libraries. The larvae were placed in a petri dish on top of the monitor, and movies of the eye movements taken with an iPhone. The latter is not inexpensive, but at least little added expense (Deveau *et al.*, 2020). The reviewer expects the parametric properties of a laptop monitor as seen from below by the larvae don’t equal those of a high-contrast drum seen from all sides, but they were good enough to identify color-vision mutations.

**Minor points.**

*Abstract: *

- Reviewer: 0.02 cycles/deg ´ 360 deg/drum =7.2 cycles/drum. Usually, one would fit an even number of cycles inside the drum. The abstract doesn’t discuss rotation rate. The reviewer presumes this is constant.
- ‘drums evoked equivalent responses with the 0.06 and 0.2 cpd drums’
  Reviewer: What does equivalent mean in this context? Equal?
- ‘The saccadic frequency in VA and CS assays was inversely proportional to age, spatial frequency and contrast of the stimulus.’
  Reviewer: Does this mean more saccades at less contrast?

*Methods:*

- ‘From a population of N=50 and N=40 larvae, n=12 larvae were randomly selected for VA and CS experiments at each timepoint for Protocol I and at 16 dpf for Protocol II, respectively. After each experiment, larvae were returned to the tank.’
  Reviewer: Were some larvae reused at several stages in development? It’s a major point in animal protocols and best to be clear. The reviewer sees no problem with this as the testing does not cause distress, and if anything, appears to interest the larvae.
- ‘CS drums ranging from 100 - 20% were generated by degrading horizontally from the lateral sides to the centre and then changing the transparency percentage of the centre of the black stripes with all retaining 0.02 cycles/degree.’
  Reviewer: This is a difficult sentence.
- ‘From another Petri dish, larval or juvenile zebrafish in embryo medium were randomly selected between all larvae in the Petri dish’
  Reviewer: ‘From another Petri dish, larval or juvenile zebrafish in embryo medium were randomly selected’

*Results:*

- ‘saccades per minute were counted’
  Fig. 1. Reviewer: I don’t see a camera in the setup. Looks like this is done manually in real time. Sounds like a visual system test for the observer too. Is there at least a clicker?
- Reviewer: In Fig. 1A it is unclear where the second light guide illumination is placed. Is the illumination uniform on the inside of the drum, or is only one hemifield illuminated?
- Reviewer: In Fig. 3, a control with ‘no drum’ might be useful. One supposes that there might be spontaneous saccades. The fish just ‘looks around’. Are the higher spatial frequency responses different from ‘no drum’?
- Reviewer: Fig. 4A. It looks like zebrafish are afternoon people, just like the reviewer.
- Legend Fig. 5 ‘Three replicates of eight larvae, n=24 larvae per pattern.’
- Reviewer: I think that is 24 measurements per pattern.
- Reviewer: In Fig. 4, is it the case among larvae that there are high performers and low performers? There is quite a spread in saccadic eye movements.

*Discussion:*

- Reviewer comment: The discussion points out that drum evokes responses at higher spatial frequencies and lower contrasts than computerized displays.
- Discussion: ‘As zebrafish became older, the saccadic frequency was decreased when spatial frequency was increased.’
  Reviewer: For all ages, saccadic frequency decreased as spatial frequency increased.

Is the rationale for developing the new method (or application) clearly explained?

Yes

Is the description of the method technically sound?

Yes

Are the conclusions about the method and its performance adequately supported by the findings presented in the article?

Yes

If any results are presented, are all the source data underlying the results available to ensure full reproducibility?

Yes

Are sufficient details provided to allow replication of the method development and its use by others?

Yes

Reviewer Expertise:

My lab has performed OKR research on zebrafish, the topic of the submission.

I confirm that I have read this submission and believe that I have an appropriate level of expertise to confirm that it is of an acceptable scientific standard.

# Response

**Published:** 2021-12-18

**Authors:** Alicia Gómez Sánchez

**Affiliations:** 22032, Ireland

Dear Dr. Nelson, thank you very much for the constructive review of our work. We really appreciate your suggestions for improvement. In the following paragraphs we address your individual comments. Minor points.

 Abstract:

- Reviewer: 0.02 cycles/deg ´ 360 deg/drum =7.2 cycles/drum. Usually, one would fit an even number of cycles inside the drum. The abstract doesn’t discuss rotation rate. The reviewer presumes this is constant.

Response: The reviewer is correct and we thank them for pointing this out so that we can clarify. To be more precise we used a 0.0277 cycles/deg. In a 360 deg/drum this equates to 10 cycles/drum. We could not use precisely 0.02, 0.04, 0.06, 0.1 and 0.2 cpd because there would be an incomplete cycle but we designed the patterns as close as possible. We have included this clarification in OKR equipment section in Methods as follows ‘The VA drums were 0.0277, 0.0416, 0.0638, 0.1111 and 0.2027 cycles per degree (cpd) were chosen based on a previous publication (15). For convenience, we simply refer to these drums as 0.02, 0.04, 0.06, 0.1 and 0.2 cpd.’ Rotation rate is constant at 100 degrees per second so as to not add another variable in the optokinetic response. This parameter is described in Drum Velocity section in Methods ‘The drum base was rotated with a constant angular velocity of 100 degrees per second’.

- ‘drums evoked equivalent responses with the 0.06 and 0.2 cpd drums’
- Reviewer: What does equivalent mean in this context? Equal?

Response: Please, see Abstract where we have changed ‘drums evoked equivalent responses with the 0.06 and 0.2 cpd drums’ to ‘0.06 and 0.2 cpd drums elicited equivalent responses with both set-ups'. They were equivalent because the averages were 7.5 and 5.3 saccades per minute for 0.06 cpd 2D and 3D-printed drum respectively and 7.9 and 5.8 saccades per minute for 0.2 cpd 2D and 3D-printed drum respectively and there was no significant difference between each pair of responses (see Fig. 3)

- ‘The saccadic frequency in VA and CS assays was inversely proportional to age, spatial frequency and contrast of the stimulus.’
- Reviewer: Does this mean more saccades at less contrast?

Response: Please see Abstract where we have corrected ‘The saccadic frequency in VA and CS assays was inversely proportional to age, spatial frequency and contrast of the stimulus’ to ‘The saccadic frequency in VA assays was inversely proportional to age and spatial frequency and in CS assays was inversely proportional to age and directly proportional to contrast of the stimulus’. We hope that it is now clarified Methods:

- ‘From a population of N=50 and N=40 larvae, n=12 larvae were randomly selected for VA and CS experiments at each timepoint for Protocol I and at 16 dpf for Protocol II, respectively. After each experiment, larvae were returned to the tank.’
- Reviewer: Were some larvae reused at several stages in development? It’s a major point in animal protocols and best to be clear. The reviewer sees no problem with this as the testing does not cause distress, and if anything, appears to interest the larvae.

Response: The reviewer is correct, some larvae were re-used. In juvenile VA and CS experiments, larvae were returned to the main system tank in compliance with our animal ethics committee approval as the assay does not cause distress and this reduces the number of animals used.  

- ‘CS drums ranging from 100 - 20% were generated by degrading horizontally from the lateral sides to the centre and then changing the transparency percentage of the centre of the black stripes with all retaining 0.02 cycles/degree.’
- Reviewer: This is a difficult sentence.

Response: We apologize if our original statement was confusing and have updated the OKR equipment Methods section to ‘CS drums ranging from 100 – 20% were generated from a 0.02 cpd pattern, maintaining the spatial frequency. The black colour of the stripes was changed to include a centre to peripheral vertical gradient generated in Powerpoint™ using the “Gradient Tool, Linear Right Option” and adjusting the transparency of the Mid Gradient Stop according to the desired contrast. For example, 40% black contrast was made by changing the transparency to 60%. (C=100%-60%=40%)’. We hope that is now clear.

- ‘From another Petri dish, larval or juvenile zebrafish in embryo medium were randomly selected between all larvae in the Petri dish’
- Suggested Reviewer Change: ‘From another Petri dish, larval or juvenile zebrafish in embryo medium were randomly selected’

Response: We agree. Please see VA and CS methods (Protocols I and II) Methods section. We have updated our original statement ‘From another Petri dish, larval or juvenile zebrafish in embryo medium were randomly selected between all larvae in the Petri dish’ to ‘From another Petri dish, larval or juvenile zebrafish in embryo medium were randomly selected’ Results:

- ‘saccades per minute were counted’
- Fig. 1. Reviewer: I don’t see a camera in the setup. Looks like this is done manually in real time. Sounds like a visual system test for the observer too. Is there at least a clicker?

Response: We appreciate this comment. We do not routinely use a camera to record the saccades. This was done manually having a timer to count the 30’’ and record the number of saccades in a lab notebook. Manual counting of saccades is routine and has been published previously (Deeti et al., 2014; Ward et al., 2020; Daly et al 2017 REFERENCE). The OKR activity of 5 dpf wild-type larvae has been completed multiple times, over many years in our lab by different researchers obtaining similar results. Thus, even though it is a manual recording, results are consistent across different observers. Furthermore, we did video-record a subset of the 16 dpf larval OKRs, which were analysed by independent observers, and to address the reviewer’s concern, they validated our results.

- Reviewer: In Fig. 1A it is unclear where the second light guide illumination is placed. Is the illumination uniform on the inside of the drum, or is only one hemifield illuminated?

Response: Reviewer is correct about this observation, the photograph in Fig. 1A does not show clearly where the second light guide is place. We have updated the legend to further explain as follows ‘The second light guide is placed on the opposite side to the first guide to uniformly illuminate the inside of the drum’.

- Reviewer: In Fig. 3, a control with ‘no drum’ might be useful. One supposes that there might be spontaneous saccades. The fish just ‘looks around’. Are the higher spatial frequency responses different from ‘no drum’?

Response: Previous researchers in our group demonstrated that when the 0.02 cpd drum was not rotated for 30 seconds (in between the clockwise and counter-clockwise rotations) that there very few or no spontaneous saccades. The sum of these were much lower than the average 29.8 saccades per minute we routinely observe in 5 dpf fish with 0.02 cpd 2D/3D-printed drum. In addition, as mentioned to Reviewer 1, we have a mutant zebrafish line with a normal OKR at the 0.02 cpd drum, but a significantly reduced number of saccades compared to siblings at the 0.2 cpd drum. This suggests these movements are predominantly responses to the drum stimuli and not spontaneous saccades when “looking around”.

- Legend Fig. 5 ‘Three replicates of eight larvae, n=24 larvae per pattern.’
- Suggested Change by Reviewer: I think that is 24 measurements per pattern

Response: We appreciate your comment. We have changed in the legend in Fig 5 ‘Three replicates of eight larvae, n=24 larvae per pattern’ to ‘24 measurements per pattern’ which is more accurate in this case.

- Reviewer: In Fig. 4, is it the case among larvae that there are high performers and low performers? There is quite a spread in saccadic eye movements.

Response: We appreciate the reviewer’s observation. Eye saccades are an innate response which presents a variability by its biological nature. Even larvae bred together under the same conditions develop at different rates, likely leading to such variability.

 Discussion:

- Discussion: ‘As zebrafish became older, the saccadic frequency was decreased when spatial frequency was increased.’
- Suggested Reviewer Change: For all ages, saccadic frequency decreased as spatial frequency increased.

Response: We appreciate the reviewer’s correction and have corrected our original state ‘As zebrafish became older, the saccadic frequency was decreased when spatial frequency was increased’ to ‘For all ages, saccadic frequency decreased as spatial frequency increased’.

# Peer review: Reviewer response for version 1

**DOI:** 10.21956/openreseurope.15007.r27957

**Published:** 2021-11-12

**Authors:** Logan Ganzen

**Affiliations:** 1 UC Berkeley, Berkeley, CA, USA

**Summary**

 The authors have presented a method article that describes the development of methods to assess zebrafish vision via the optokinetic response (OKR). Visual acuity (VA) and contrast sensitivity (CS) was assessed by utilizing either a rotating 3D printed striped drum or a 2D paper printed stripe pattern around larval and juvenile zebrafish. By varying stripe spatial frequency, contrast, and chamber illuminance, the authors are able to discern differences in the OKR between conditions. The authors present their method and findings as a low-cost and easy way to reliably assess fine details of zebrafish OKR behavior without needing to purchase expensive computer-assisted technologies. Overall, the article is well-written and scientifically sound. However, there are some addressable concerns with the study.

**General Comments**

1. One of the justifications provided by the authors for the development of these OKR methods was to potentially “identify more subtle defects in zebrafish vision”. While the authors demonstrate that the described methods have the capacity to identify changes in OKR behavior of healthy zebrafish, no direct evidence is provided to demonstrate that the authors’ methods are capable of resolving defects in zebrafish vision potentially arising from a disease state. The authors should consider providing an example that one or more of their OKR methods can discern a subtle visual defect in an unhealthy zebrafish as compared to a healthy zebrafish. One potential experiment would be to acutely treat zebrafish larvae with ethanol and demonstrate some of the authors’ methods can discern defects in OKR behavior. Previous work has demonstrated that acute EtOH treatment alters the light intensity threshold of zebrafish larvae[1-2]. More recent work has also demonstrated that the GABA­ _A_ antagonist picrotoxin can modulate the zebrafish OKR[3]. Pharmacological treatment to alter the OKR could potentially be done quickly and without much difficulty.
2. The authors have gone through the trouble to create a few 3D-printed striped barrels of varying cpd and demonstrated that they are no less effective than their 2D-printed paper barrels in Fig 3. The authors use the “standard 0.02 cpd 3D printed barrel” in Fig. 4 and switch to the 2D barrels for the remaining figures. The authors do not provide much discussion regarding the pros and cons of using either the 3D or 2D barrels. Are the 3D barrels limited in cpd due to the manufacturing process? Is it possible to generate 3D barrels for CS testing? As it stands, the addition of the 3D barrel in the manuscript is somewhat awkward since all of the authors’ experiments could have been accomplished with the 2D barrels alone. The authors should critically discuss and justify the use of 3D barrels.
3. The authors have provided the patterns for the 2D barrels in Fig 2. If the manuscript page is printed on a Letter or A4 sheet, are these patterns the proper dimensions to cut and form into a usable barrel? The authors should describe this. For reproducibility of this method between labs to be accurate, the authors should deposit a .svg. .psd, or some other file that contains the pattern on the correct sheet dimension. Additionally, the authors should deposit the files for the 3D-printed barrels as well. The authors could discuss the potentials for the standardization of manual OKR stimuli if these resources are available as open-source to the community.

**Specific Concerns (page # as in pdf)**

- Abstract:
  N/A
- Introduction:
  Page 3: “One approach to more thoroughly vision evaluations…” Grammar.
- Page 3: See general comment 2 on 3D barrel justification.
- Methods:
  Page 4: “9% methylcellulose”. Many zebrafish studies publish immobilization in a methylcellulose concentration of around 3%. Would a more viscous solution at 9% potentially alter the saccades per minute statistic measured in the OKR experiments compared with a 3% solution? The authors mention difficulty immobilizing zebrafish beginning at 16 dpf which may call for a higher viscosity solution, but larval fish should sufficiently be immobilized in 3% methylcellulose. The authors should elaborate on this concentration.
- Results:
  Page 9 (and onward): The authors describe values in the text as X number of saccades per minute but do not clearly define if that statistic is an average or a median value of their distribution. Additionally, authors should specify the standard deviation for each statistic they describe.
  Page 9: Figure 3 (and onward): The authors describe the error bars as 1 S.D. but should also explicitly state that the midline of the error bars represents the distribution average (if this is true).
  Page 9/10: Figure 4a. Authors state that the OKR at the 127.5 hpf time point was significantly different from the 121.5 hpf time point on page 9, but Fig. 4a does not reflect this statistical test.
  Page 10: Figure 4a. The light cycle diagram at the top of the figure is probably incorrect. The authors likely meant to indicate that the light turned on at 7:30am, not 7:30pm. The diagram format and alignment are also somewhat confusing.
  Page 10: Figure 4b (and onward). Since the authors have described a time-of-day dependency of the OKR at 5 dpf in Fig 4a, what time of day was the Luminance experiment conducted at, and was the time of day kept consistent for all subsequent experiments and figures? Authors should clarify what time of day the other experiments were performed at.
  Page 10: Figure 5a. The extreme outlier in the 0.1 cpd group may cause confusion with the statistical stars due to its location. Authors may wish to address this.
  Page 12: Figure 6a: The color scheme provided makes it very difficult to discern the colors of 6 dpf and 12 dpf. Authors may wish to consider.
  Page 11/12: Figure 6b. The authors state on page 11 that “In 5 dpf zebrafish, there was no significant difference in OKR using Protocol I or II for 0.2 cpd pattern (Figure 6B)” but do not actually show the data or statistical test for protocol 2 in the figure. Additionally, it appears that the authors have copied the 5 dpf and 16dpf protocol 1 data from Fig. 6a and used it again in Fig. 6b without being explicit that these are the same datasets. This should be made clear to avoid confusion that arises with the statistics being performed (The authors could maintain the color scheme of each protocol 1 dataset from Fig. 6a in Fig. 6b). While the protocol 1 experiment creates paired data which is appropriate for a one-way repeated ANOVA, protocol 2 creates unpaired data which should not be used with the repeated measures one-way ANOVA described in the figure legend. Comparing protocol 1 and protocol 2 datasets should be done in an unpaired fashion.
  Page 13: Figure 7a and b: See previous comments with Figure 6a. Any data used from 7a into 7b should be explicitly stated. Issues with the paired statistical test on unpaired data are present as in 6a and 6b. Should independent protocol 2 data with 100% contrast be added to Fig 7b. for completeness? Fig. 7a appears to be somewhat blurry compared with other figures. 
- Discussion:
  Consider elaborating on the 3D barrel as mentioned in general comment 2.

Is the rationale for developing the new method (or application) clearly explained?

Yes

Is the description of the method technically sound?

Yes

Are the conclusions about the method and its performance adequately supported by the findings presented in the article?

Partly

If any results are presented, are all the source data underlying the results available to ensure full reproducibility?

Yes

Are sufficient details provided to allow replication of the method development and its use by others?

Partly

Reviewer Expertise:

My area of research is generally in visual neuroscience. My doctoral research involved the development of zebrafish behavioral assays to perform drug screening on zebrafish models of disease.

I confirm that I have read this submission and believe that I have an appropriate level of expertise to confirm that it is of an acceptable scientific standard, however I have significant reservations, as outlined above.

## References

[1] : Effects of ethanol on photoreceptors and visual function in developing zebrafish. *Invest Ophthalmol Vis Sci* .2006;47(10) : 10.1167/iovs.05-0971 4589-97 10.1167/iovs.05-0971 17003456PMC2408731

[2] : Effects of embryonic exposure to ethanol on zebrafish visual function. *Neurotoxicology and Teratology* .2002;24(6) : 10.1016/S0892-0362(02)00319-7 759-766 10.1016/S0892-0362(02)00319-7 12460658

[3] : Modulation of the zebrafish optokinetic reflex by pharmacologic agents targeting GABAA receptors. *Neuroscience Letters* .2018;671: 10.1016/j.neulet.2018.01.055 33-37 10.1016/j.neulet.2018.01.055 PMC588974429410359

# Response

**Published:** 2021-12-18

**Authors:** Alicia Gómez Sánchez

**Affiliations:** 22032, Ireland

Dear Prof. Ganzen, thank you very much for the constructive review of our work. We really appreciate your suggestions for improvement. In the following paragraphs we address your individual comments. General Comments

1. One of the justifications provided by the authors for the development of these OKR methods was to potentially “identify more subtle defects in zebrafish vision”. While the authors demonstrate that the described methods have the capacity to identify changes in OKR behavior of healthy zebrafish, no direct evidence is provided to demonstrate that the authors’ methods are capable of resolving defects in zebrafish vision potentially rising from a disease state. The authors should consider providing an example that one or more of their OKR methods can discern a subtle visual defect in an unhealthy zebrafish as compared to a healthy zebrafish. One potential experiment would be to acutely treat zebrafish larvae with ethanol and demonstrate some of the authors’ methods can discern defects in OKR behavior. Previous work has demonstrated that acute EtOH treatment alters the light intensity threshold of zebrafish larvae[1-2]. More recent work has also demonstrated that the GABA­A antagonist picrotoxin can modulate the zebrafish OKR[3]. Pharmacological treatment to alter the OKR could potentially be done quickly and without much difficulty.

Response: We appreciate this comment. As the reviewer says, we show that the methods to assess VA and CS in zebrafish larvae are effective. Inherently, assays that more sensitively assess vision can uncover more subtle defects in vision. The experiments suggested by the reviewer are indeed interesting, but there is a reasonable chance that these treatments could affect even the standard OKR and therefore would not achieve the objective. However, we have assessed VA and CS in two zebrafish knockout lines. In one case, there was no change compared to siblings. In another, a significant VA/CS defect was observed only in the mutant compared to siblings with the highest spatial frequency and lowest contrast sensitivity. These pieces of data are being included in other manuscripts (one under review) wherein those genes are the focus of the manuscripts. Therefore, it is best to include that VA/CS data in those manuscripts. In summary, the assays will not always uncover more subtle defects in vision, but fundamentally they can because they require better visual acuity or contrast sensitivity. We have an example in a mutant line and this will be published shortly corroborating these methods. However, the main findings from this manuscript strand alone.

1. The authors have gone through the trouble to create a few 3D-printed striped barrels of varying cpd and demonstrated that they are no less effective than their 2D-printed paper barrels in Fig 3. The authors use the “standard 0.02 cpd 3D printed barrel” in Fig. 4 and switch to the 2D barrels for the remaining figures. The authors do not provide much discussion regarding the pros and cons of using either the 3D or 2D barrels. Are the 3D barrels limited in cpd due to the manufacturing process? Is it possible to generate 3D barrels for CS testing? As it stands, the addition of the 3D barrel in the manuscript is somewhat awkward since all of the authors’ experiments could have been accomplished with the 2D barrels alone. The authors should critically discuss and justify the use of 3D barrels.

Response: Thank you for your question. Here, we present two affordable set-ups to measure visual behaviour in zebrafish. 2D drums have the advantage of being printed inexpensively, however, they are printed on cardboard which can be broken or deformed easily. 3D drums, by contrast, are made of PLA, which makes the drum rigid, harder to break and thus, longer lasting. Nevertheless, an external company manufactures the 3D drums, so the cost price is higher (less than 100€/drum) than 2D drums but being still considerably more affordable than an automated OKR. We started with the2D-printed drums as they could quickly and affordably be generated in-house. For sustainable, long-term use of the same drums we consider the 3D drums a better option and therefore we considered it important to prove that both set-ups work in practice and yield equivalent results which we show with the VA drums. We have clarified this point at the end of the introduction as follows ‘Here, we describe two simple and affordable methods to assess VA and CS in zebrafish using either 2D and 3D printed striped patterns/drums to quantify OKR, VA and CS in larval and juvenile zebrafish’. We also discuss this point in the Discussion section with the heading Affordability and Accessibility which has been updated to state: ‘While 2D-printed can be easily printed on cardboard, they are less durable so can be used for pilot experiments. However, if researchers prefer a more durable option, 3D-printed drums are still an affordable option (less than 100€ (per drum) yielding equivalent results as 2D-printed drums’

1. The authors have provided the patterns for the 2D barrels in Fig 2. If the manuscript page is printed on a Letter or A4 sheet, are these patterns the proper dimensions to cut and form into a usable barrel? The authors should describe this. For reproducibility of this method between labs to be accurate, the authors should deposit a .svg. .psd, or some other file that contains the pattern on the correct sheet dimension. Additionally, the authors should deposit the files for the 3D-printed barrels as well. The authors could discuss the potentials for the standardization of manual OKR stimuli if these resources are available as open-source to the community.

Response: We appreciate your suggestions. Patterns in Fig 2 are ready to cut and build if printed on A4 sheets. For reproducibility purposes, please see OKR equipment Methods section where we provide company contact details (projects@materialise.co.uk) and all details necessary (drum dimensions: 3.4 cm high and 6 cm in diameter; desired cycles per degree: 0.02, 0.04, 0.06, 0.1 or 0.2 cpd and stripes colour: black and white) to get 3D drums manufactured as ours. The files to print the 3D drums were generated by the commercial supplier and we do not have access to them to deposit them as queried. We agree that standardization of OKR stimuli (indeed the overall assay) is a worthy goal and therefore we have included all the details of the patterns that we have in the Methods and have considered several causes of variability in OKR assays in the Discussion. Specific Concerns (page # as in pdf) 

- Introduction: Page 3: “One approach to more thoroughly vision evaluations…” Grammar.

Response: We agree and have changed our original statement: ‘One approach to more thoroughly vision evaluations…’ to ‘One approach to assessing vision more thoroughly is to vary the optokinetic stimulation’.

- Page 3: See general comment 2 on 3D barrel justification.

Response: This point was also addressed above. We have updated the Affordability and Accessibility section in the Discussion. We have discussed the differences between 2D and 3D-printed drums as follows ‘While 2D-printed can be easily printed on cardboard, they are less durable so can be used for pilot experiments. However, if researchers prefer a more durable option, 3D-printed drums are still an affordable option (less than 100€ (per drum) yielding equivalent results as 2D-printed drums’

- Methods: Page 4: “9% methylcellulose”. Many zebrafish studies publish immobilization in a methylcellulose concentration of around 3%. Would a more viscous solution at 9% potentially alter the saccades per minute statistic measured in the OKR experiments compared with a 3% solution? The authors mention difficulty immobilizing zebrafish beginning at 16 dpf which may call for a higher viscosity solution, but larval fish should sufficiently be immobilized in 3% methylcellulose. The authors should elaborate on this concentration.

Response: We appreciate this comment and the need for researchers to elaborate on the methylcellulose used in their studies. Previous studies have also used more than 3% methylcellulose (i.e. 6% or 9%) (e.g. Brockerhoff et al, 20061; Zou et al, 20102; Deeti et al 20143; Daly et al, 20174; Ward et al, 20205; Ward et al, 20206) (see references at the end) obtaining a similar number of saccades at 5 dpf as we report here. Notably, there are different grades of methylcellulose with different viscosities (cP values). We routinely used methylcellulose of 25 cP (Sigma M6385) in our studies. We do not have ethical approval to place zebrafish animals older than 131 hpf in methylcellulose >9%.

 Results:

 Page 9 (and onward): The authors describe values in the text as X number of saccades per minute but do not clearly define if that statistic is an average or a median value of their distribution. Additionally, authors should specify the standard deviation for each statistic they describe. Response: We have clarified it in Statistical analysis methods section as follows ‘All saccades per minute values described in this manuscript are a group average’. The standard deviation has been included for each statistic value (saccades per minute group average) described in the manuscript text. Page 9: Figure 3 (and onward): The authors describe the error bars as 1 S.D. but should also explicitly state that the midline of the error bars represents the distribution average (if this is true). Response: We apologise for this oversight and included a statement in the legend of Figure 3 and onward as follows ‘Midline of error bars represents the group average’ Page 9/10: Figure 4a. Authors state that the OKR at the 127.5 hpf time point was significantly different from the 121.5 hpf time point on page 9, but Fig. 4a does not reflect this statistical test. Response: Figure 4a has been updated and now reflects the statistical difference between 121.5 and 127.5 hpf.

 Page 10: Figure 4a. The light cycle diagram at the top of the figure is probably incorrect. The authors likely meant to indicate that the light turned on at 7:30am, not 7:30pm. The diagram format and alignment are also somewhat confusing. Response: We thank the reviewer for spotting this error. The light cycle diagram has been modified for better understanding. We hope it is now clearer.

 Page 10: Figure 4b (and onward). Since the authors have described a time-of-day dependency of the OKR at 5 dpf in Fig 4a, what time of day was the Luminance experiment conducted at, and was the time of day kept consistent for all subsequent experiments and figures? Authors should clarify what time of day the other experiments were performed at. Response: We appreciate this comment and have included what time of day the experiments from Fig 4b and onwards were performed. Experiment from Fig. 4b was done at 125 hpf (please see updated The zebrafish larval OKR response is modulated by time of day and luminance levels in Results section (Pag. 11)) Experiment from Fig. 5a was done at 123 hpf which was included in the original manuscript (please see ‘The 2D/3D-printed striped patterns enable discrimination of VA and CS in larval zebrafish’ in Results section (Pag. 11)) Experiment from Fig. 5b was done at 125 hpf which was included in the original manuscript (please see ‘The 2D/3D-printed striped patterns enable discrimination of VA and CS in larval zebrafish’ in Results section (Pag. 11)) Experiment from Fig. 6a was done at 123 hpf (please see updated ‘The zebrafish VA response shows age-dependent variations’ in Results section (Pag. 11)) Experiment from Fig. 6b was done at 123 hpf (please see updated ‘The zebrafish VA responses show age-dependent variations’ in Results section (Pag. 11 and 14)). Experiment from Fig. 7a was done at 125 hpf (please see updated ‘The zebrafish CS response shows age-dependent variations’ in Results section (Pag. 11)) Experiment from Fig. 7b was done at 125 hpf (please see updated ‘The zebrafish CS responses show age-dependent variations’ in Results section (Pag. 11 and 14))

 Page 10: Figure 5a. The extreme outlier in the 0.1 cpd group may cause confusion with the statistical stars due to its location. Authors may wish to address this. Response: We agree and have updated Figure 5a accordingly.

 Page 12: Figure 6a: The color scheme provided makes it very difficult to discern the colors of 6 dpf and 12 dpf. Authors may wish to consider. Response: We have changed the color scheme for all groups in Figure 6 and 7 which it is now more visual.

 Page 11/12: Figure 6b. The authors state on page 11 that “In 5 dpf zebrafish, there was no significant difference in OKR using Protocol I or II for 0.2 cpd pattern (Figure 6B)” but do not actually show the data or statistical test for protocol 2 in the figure. Additionally, it appears that the authors have copied the 5 dpf and 16dpf protocol 1 data from Fig. 6a and used it again in Fig. 6b without being explicit that these are the same datasets. This should be made clear to avoid confusion that arises with the statistics being performed (The authors could maintain the color scheme of each protocol 1 dataset from Fig. 6a in Fig. 6b). While the protocol 1 experiment creates paired data which is appropriate for a one-way repeated ANOVA, protocol 2 creates unpaired data which should not be used with the repeated measures one-way ANOVA described in the figure legend. Comparing protocol 1 and protocol 2 datasets should be done in an unpaired fashion. Response: We wish to clarify that the bottom right corner of the Fig 6B graph for 5 dpf larvae shows there is no significant difference for the 0.2 drum between the protocols. We thank the reviewer for the opportunity to clarify this point. We do re-use the from 5 and 16 dpf Protocol I datasets from Fig 6a in Fig 6B (which include new data for Protocol II) as conditions were the same and we have a limited number of approved fish for these experiments under our ethical approval. We have clarified this in the revised manuscript updating The zebrafish VA responses shows age-dependent variations Results section (Pag. 11) as follows ‘In Figure 6b, datasets from 5 and 16 dpf groups for Protocol I are from the same groups in Figure 6a’. We thank the reviewer for spotting this incorrect sentence in the legend. The data from Figure 6B was analysed using one-way ANOVA unpaired measures and Tukey’s multiple comparisons test and this has been revised in the Statistical Analysis methods section and in the figure legend. Page 13: Figure 7a and b: See previous comments with Figure 6a. Any data used from 7a into 7b should be explicitly stated. Issues with the paired statistical test on unpaired data are present as in 6a and 6b. Should independent protocol 2 data with 100% contrast be added to Fig 7b. for completeness? Fig. 7a appears to be somewhat blurry compared with other figures. Response: We have clarified this, updating The zebrafish CS responses shows age-dependent variations Results section (Page. 11/Page.14) as follows ‘In Figure 7b, datasets from 5 and 16 dpf groups for Protocol I are from the same groups in Figure 7a’ (Page 14). We thank the reviewer for spotting this incorrect sentence in the legend. The data from Figure 7B was analysed using one-way ANOVA unpaired measures and Tukey’s multiple comparisons test and this has been revised in the Statistical Analysis methods section and in the figure legend. Protocol II refers to when the larvae are naïve to a stimulus whereas in Protocol I the larvae move form highest contrast (100%) to lower contrast stimuli. Therefore, the 100% contrast is actually the naïve response in Protocol II. The resolution of Fig 7a may have been reduced in the process of generating a PDF. We have revised the figure to be of higher-resolution. Discussion:

 Consider elaborating on the 3D barrel as mentioned in general comment 2. Response: This was addressed earlier and is revised in the Discussion section.

 References: 

 1. Brockerhoff SE. Measuring the optokinetic response of zebrafish larvae. Nat Protoc. 2006;1(5):2448-51. doi: 10.1038/nprot.2006.255. PMID: 17406490.

 2. Zou, S., Yin, W., Zhang, M., Hu, C., Huang, Y., Hu, B. Using the optokinetic response to study visual function of zebrafish. J. Vis. Exp. (36), e1742, doi:10.3791/1742 (2010).

 3. Deeti S, O'Farrell S, Kennedy BN. Early safety assessment of human oculotoxic drugs using the zebrafish visualmotor response. J Pharmacol Toxicol Methods. 2014 Jan-Feb;69(1):1-8. doi: 10.1016/j.vascn.2013.09.002. Epub 2013 Sep 30. PMID: 24091134.

 4. Daly, Conor, et al. "A brain-derived neurotrophic factor mimetic is sufficient to restore cone photoreceptor visual function in an inherited blindness model." Scientific reports 7.1 (2017): 1-18.

 5. Ward R, Ali Z, Slater K, Reynolds AL, Jensen LD, Kennedy BN. Pharmacological restoration of visual function in a zebrafish model of von-Hippel Lindau disease. Dev Biol. 2020 Jan 15;457(2):226-234. doi: 10.1016/j.ydbio.2019.02.008. Epub 2019 Feb 27. PMID: 30825427.

 6. Ward R, Kaylor JJ, Cobice DF, Pepe DA, McGarrigle EM, Brockerhoff SE, Hurley JB, Travis GH, Kennedy BN. Non-photopic and photopic visual cycles differentially regulate immediate, early, and late phases of cone photoreceptor-mediated vision. J Biol Chem. 2020 May 8;295(19):6482-6497. doi: 10.1074/jbc.RA119.011374. Epub 2020 Apr 1. PMID: 32238432; PMCID: PMC7212626.
